# Development of Novel Glyphosate-Tolerant *Japonica* Rice Lines: A Step Toward Commercial Release

**DOI:** 10.3389/fpls.2016.01218

**Published:** 2016-08-30

**Authors:** Ying Cui, Shuqing Huang, Ziduo Liu, Shuyuan Yi, Fei Zhou, Hao Chen, Yongjun Lin

**Affiliations:** ^1^National Key Laboratory of Crop Genetic Improvement and National Center of Plant Gene Research, Huazhong Agricultural UniversityWuhan, China; ^2^National Key Laboratory of Agricultural Microbiology, Huazhong Agricultural UniversityWuhan, China

**Keywords:** 5-enolpyruvylshikimate-3-phophate synthase, I. variabilis-EPSPS^*^, glyphosate-tolerant, *Isoptericola variabilis*, rice, commercial release

## Abstract

Glyphosate is the most widely used herbicide for its low cost and high efficiency. However, it is rarely applied directly in rice field due to its toxicity to rice. Therefore, glyphosate-tolerant rice can greatly decrease the cost of rice production and provide a more effective weed management strategy. Although, several approaches to develop transgenic rice with glyphosate tolerance have been reported, the agronomic performances of these plants have not been well evaluated, and the feasibility of commercial production has not been confirmed yet. Here, a novel glyphosate-tolerant gene cloned from the bacterium *Isoptericola variabilis* was identified, codon optimized (designated as I. variabilis-EPSPS^*^), and transferred into Zhonghua11, a widely used *japonica* rice cultivar. After systematic analysis of the transgene integration via PCR, Southern blot and flanking sequence isolation, three transgenic lines with only one intact I. variabilis-EPSPS^*^ expression cassette integrated into intergenic regions were identified. Seed test results showed that the glyphosate tolerance of the transgenic rice was about 240 times that of wild type on plant medium. The glyphosate tolerance of transgenic rice lines was further evaluated based on comprehensive agronomic performances in the field with T_3_ and T_5_generations in a 2-year assay, which showed that they were rarely affected by glyphosate even when the dosage was 8400 g ha^−1^. To our knowledge, this is the first demonstration of the development of glyphosate-tolerant rice lines based on a comprehensive analysis of agronomic performances in the field. Taken together, the results suggest that the selected glyphosate-tolerant rice lines are highly tolerant to glyphosate and have the possibility of commercial release. I. variabilis-EPSPS^*^ also can be a promising candidate gene in other species for developing glyphosate-tolerant crops.

## Introduction

Rice is a major cereal crop and feeds more than half of the world population. It is important to enhance rice production to meet the increasing demand for food. But rice production is currently faced with challenges, such as the reduction of arable land, the decrease of farming labor force, and the shortage of water resources (Van Nguyen and Ferrero, 2006). Due to these factors, there is a shift from traditional transplanting to direct seeding of rice in many areas (Farooq et al., 2011). The ratio of direct seeded rice has reached 26% in south Asia and 23% in the world (Rao et al., 2007). However, in the direct seeding system, both the yield and quality of rice are severely affected by weeds (Rao et al., 2007; Akbar et al., 2011). It is estimated that the yield loss due to uncontrolled weeds is over 80% in direct seeding system (Singh et al., 2011; Chauhan, 2013). In traditional agriculture, weed management mainly depends on hand weeding and water management, which is time- and labor-consuming. Compared with hand weeding, herbicide management requires less labor, and funding and is more efficient, which makes it the most widely adopted approach in rice cultivation (Akbar et al., 2011; Chauhan, 2013; Antralina et al., 2015; Chauhan et al., 2015).

Glyphosate was developed as a herbicide by Monsanto in the 1970s. Because this chemical disrupts aromatic amino acid synthesis by specifically inhibiting the enzyme 5-enolpyruvylshikimate-3-phosphate synthase (EPSPS) of the shikimate pathway which is found only in plants, bacteria, fungi, and apicomplexan parasites, it is highly active to a wide range of weeds but has little effect on mammals (Steinrücken and Amrhein, 1980; Herrmann, 1995; Roberts et al., 1998; Richards et al., 2006). The benefits of glyphosate also include low cost and easy degradation in the environment (Duke and Powles, 2008). With these advantages, glyphosate is still the most widely used herbicide after over 40 years of application. Although, the advantages of glyphosate are obvious, it is rarely applied directly in rice field due to its toxicity to rice. Therefore, the development of glyphosate-tolerant rice will greatly reduce the production cost and promote the development of direct seeded rice.

There have already been some reports about glyphosate-tolerant rice, such as transgenic rice with *G6* gene, *MdEPSPS* mutant, *OsEPSPS* mutant, *CP4-EPSPS, VvEPSPS* mutant, and *AroA*_*J*.*sp*_ (Zhao et al., 2011; Tian et al., 2013, 2015; Chandrasekhar et al., 2014; Deng et al., 2014; Chhapekar et al., 2015; Yi et al., 2016). Transgenic rice in these reports presented normal morphology 7 or 10 d after glyphosate treatment, suggesting that these transgenic rice plants are tolerant to glyphosate. As agronomic performances of these transgenic rice plants under glyphosate treatment were not evaluated, the feasibility of them for commercial production has not been confirmed. Studies on other glyphosate-tolerant crops have implied the possibility of yield loss caused by the effect of late glyphosate applications on reproductive tissues (Yasuor et al., 2007). Therefore, it is necessary to ensure that the agronomic performance of the glyphosate-tolerant crops is not affected by the late application of high-dosage glyphosate.

*I. variabilis*-EPSPS is a novel and special glyphosate-tolerant EPSPS isolated from the bacterium *Isoptericola variablilis* (Yi et al., 2015). The amino acid sequence and structure of *I. variabilis*-EPSPS are similar to that of classI EPSPSs, but the enzymatic activity is somewhat like that of classII EPSPSs. This enzyme shows high tolerance to glyphosate when expressed in *Escherichia coli*, but its function in plants and the potential to be used for developing transgenic crops remain unknown. Here, we optimized the original nucleotide sequence of *I. variabilis-EPSPS* according to codon bias in rice. A plant transformation vector was constructed based on the codon optimized sequence and transferred into the *japonica* rice cultivar Zhonghua11 by *Agrobacterium*-mediated transformation. The glyphosate tolerance of the regenerated rice plants was evaluated by both plant medium culturing and field assay after molecular verification. Finally, it was concluded that the selected transgenic rice plants were highly glyphosate-tolerant and I. variabilis-EPSPS^*^ (codon-optimized *I. variabilis-EPSPS*) can be a valuable candidate gene for the development of glyphosate-tolerant rice. Currently, I. variabilis-EPSPS^*^ has been provided to the major institutes of China for breeding of transgenic maize, canola, soybean and cotton, and it can be expected that I. variabilis-EPSPS^*^ will play an important role in the development of transgenic crops in China.

## Materials and methods

### Codon optimization and construction of plant transformation vector

The native *I. variabilis-EPSPS* from *I, variabilis* with a length of 1374 bp codes for a protein of 458 amino acids (Yi et al., 2015). The sequences of *I. variabilis-EPSPS* and a chloroplast targeting signal peptide coding sequence (*ctp*) from *Arabidopsis thaliana EPSPS* (Genebank: X06613.1) were analyzed with GenScript Rare Codon Analysis tool and optimized based on codon bias in rice. The optimized sequences were designated as I. variabilis-EPSPS^*^ and ctp^*^ respectively. The two sequences were fused together and synthesized by TaKaRa Biotech Co., Ltd. (Dalian, China). The binary vector pCAMBIA1300 (provided by the Center for the Application of Molecular Biology in International Agriculture, Australia) was digested with restriction endonucleases *Xho*I and *Eco*RI to delete the hygromycin phosphotransferase gene (*hpt*), then the vector band with a length of 7 kb was recovered, converted from sticky ends to blunt ends and circularized to generate a modified binary vector p130. Subsequently, the maize *Ubiquitin1* promoter and the fused sequence of ctp^*^ and I. variabilis-EPSPS^*^ were inserted into the multiple cloning site of p130 sequentially, and the final plant transformation vector was named as pU130 (*Ubi*-1: I.variabilis-EPSPS^*^: *35S polyA*). The vector was introduced into *Agrobacterium tumefaciens* (*EHA*105) by electroporation, and the recombinant *EHA*105 was designated as *EHA*105 (I. variabilis-EPSPS^*^).

### *Agrobacterium*-mediated transformation

An elite *japonica* rice cultivar Zhonghua11 was selected to be the receptor in *Agrobacterium*-mediated transformation. The transformation was carried out according to the method of Hiei et al. (1994), except that the resistant calli were selected with 200 mg L^−1^ glyphosate in a period of 5 weeks.

### PCR analysis of transgenic rice

Genomic DNA was extracted from transgenic rice, and amplified with primer I. variabilis-EPSPS^*^-F (5′-ATTAGCGCTAGGGACGTGAG-3′) and I. variabilis-EPSPS^*^-R (5′-ATACGCTCCCACATCCTGTC-3′). PCR was carried out with 50 ng of rice genomic DNA, 2 μL 10 × PCR buffer (Mg^2+^ plus), 0.4 μL dNTP (10 mM), 0.3 μL of each I. variabilis-EPSPS^*^-F (10 μM) and I. variabilis-EPSPS^*^-R (10 μM), 1 U *Taq* DNA polymerase in a total volume of 20 μL. The PCR conditions were 94°C for 5 min, then 30 cycles of 94°C for 30 s, 58°C for 30 s, 72°C for 40 s, and finally 72°C for 10 min.

### Assay of glyphosate tolerance in T_0_ transgenic plants

All the T_0_ transgenic plants including both PCR positive and PCR negative plants were transplanted into soil. Two weeks later, glyphosate solution at the concentration of 3000 mg L^−1^ and supplemented with 0.5% (v/v) Tween 20 was sprayed over these transgenic plants. Then the growth of these plants was carefully and continuously observed.

### Southern blot analysis

Genomic DNA was isolated from transgenic and non-transgenic rice plants by CTAB method (Murray and Thompson, 1980). Southern blot analysis was performed with DIG-labeled non-radioactive detection system. 0.3 ng transformation vector pU130 (*Ubi*-1: I. variabilis-EPSPS^*^:*35S polyA*) and 10 μg rice genomic DNA were digested with restriction endonuclease *Hin*dIII, and then separated on a 0.8% agarose gel by electrophoresis and capillary transferred onto the positively charged nylon membrane. DIG-labeled probe was prepared by the PCR-labeling method with primer I. variabilis-EPSPS^*^-F and I. variabilis-EPSPS^*^-R as mentioned above. The probe labeling, prehybridization, hybridization, and chemiluminescent detection were conducted according to the DIG application manual provided by Roche Diagnostics GmbH (Mannheim, Germany).

### Isolation of the flanking sequence of T-DNA

The flanking sequence of T-DNA was isolated by inverse PCR to analyze the integration feature of I. variabilis-EPSPS^*^ expression cassette on rice genome. One microgram rice genomic DNA was digested with restriction endonuclease *Hin*dIII or *Sac*I, and then self-ligated with T4 DNA ligase. Subsequently, nested PCR was carried out, in which 0.5 μL of the ligation product was amplified with primer Ubi-1 (5′-ACTGTAGAGTCCTGTT GTCAAAATACTCAA-3′) and IVA-1 (5′- TACGCCGACCATCGCATGGCTACATCCGCC -3′) for the first round of PCR and 0.5 μL of the first round PCR product was amplified with primer Ubi-2 (5′-TAGATAAACTGCACTT CAAACAAGTGTGAC-3′) and IVA-2 (5′- TTCGACAGGATGTGGGAGCGTATGCTCGCT -3′) for the second round of PCR. The two rounds of PCR were performed following the amplification system for *KOD-Plus* DNA polymerase (TOYOBO Co., Ltd., Osaka, Japan) provided by the manufacturer. Then, products of the second round PCR were separated by electrophoresis, recovered and sequenced. The sequences were analyzed by performing a BLAST search in NCBI database and Rice Genome Annotation Project database to investigate the integration feature of I. variabilis-EPSPS^*^ on rice genome.

### Selection of T_1_ homozygous transgenic lines

The seeds of T_1_ transgenic lines were harvested separately, and over 200 seeds from each lines were dehulled, sterilized, and subsequently transferred into 1/2 MS medium containing 0 or 30 mg L^−1^ glyphosate, and cultured at 28°C with 16 h light/8 h dark for 7 d, aiming to confirm the I. variabilis-EPSPS^*^ homozygous lines. The total tested seeds and sprouting seeds of the heterozygous lines were counted to evaluate the inheritance pattern of I. variabilis-EPSPS^*^.

### Analysis of glyphosate tolerance of T_2_ homozygous transgenic lines on plant medium

Glyphosate tolerance of homozygous T_2_ progenies of the transgenic lines and wild type Zhonghua11 was assayed by culturing on 1/2 MS medium containing different concentrations of glyphosate. The glyphosate concentrations for wild type Zhonghua11 included 0, 1, 2, 3, 4, 6, 8, and 10 mg L^−1^, and 30 seeds of Zhonghua11 were tested for each treatment. The glyphosate concentrations for homozygous transgenic progenies included 0, 30, 90, 180, 270, 360, 450, and 540 mg L^−1^, and 80 seeds of each transgenic line for each concentration were separately tested. All the seeds were cultured at 28°C with 16 h light/8 h dark for 10 d, then the heights of the seedlings cultured with different concentrations of glyphosate were measured and the mean height for each treatment was calculated. The final data were expressed as relative heights by comparing the mean height of each transgenic line for each concentration with their mean heights at 0 mg L^−1^ glyphosate. Then, the concentrations of glyphosate and the relative heights of Zhonghua11 and the three transgenic lines under different treatments were fitted to a sigmoidal logistic model (Equation 1) to get dose-response curves. The glyphosate concentrations giving a relative height of 50% (I_50_) for homozygous transgenic lines and wild type Zhonghua11 were compared to evaluate the glyphosate tolerance of homozygous transgenic lines.

(1) $$\begin{array}{l} \text{Y}=\text{C}+\frac{D-C}{1+{\left(\frac{X}{A}\right)}^{B}} \end{array}$$

In Equation 1, Y is the relative height (%); *X* is the glyphosate concentration (mg L^−1^); C is the lower limit calculated with the formula C = 0.5 cm actual mean height at 0 mg L^−1^ glyphosate (cm) × 100%, in which 0.5 cm is an empirical value (the heights of wild type Zhonghua11 seedlings on the medium containing a concentration of glyphosate which could completely inhibit the growth of Zhonghua11 after germination); *D* is the upper limit, which is set as 100% in this research; *A* is the glyphosate concentration giving a relative height of 50%; and *B* is the slope of the curve around A (Seefeldt et al., 1995).

### Northern blot and western blot

Total RNA was extracted with Trizol reagent (TransGen Biotech Co., Ltd., Beijing, China) from T_3_ homozygous transgenic lines and wild type Zhonghua11 at seedling stage. Ten micrograms RNA was separated on a 1.2% formaldehyde/MOPS gel by electrophoresis and capillary transferred onto the positively charged nylon membrane. The probe for Northern blot was the same as the probe for Southern blot, and the prehybridization, hybridization, and chemiluminescent detection were carried out following the DIG application manual provided by Roche Diagnostics GmbH (Mannheim, Germany).

*I. variabilis*-EPSPS tagged with glutathione-S-transferase (GST) was expressed in *E. coli* BL21 (DE3) and was purified using Glutathione Sepharose 4B (GE Healthcare Bio-Sciences AB, Uppsala, Sweden). Then the recombinant protein was injected into rabbits to develop polyclonal antibodies, which was finished by YouLong Biotech. Co., Ltd. (Shanghai, China). Total protein of the selected rice plants at T_3_ generation was isolated with extraction buffer containing 20 mM Tris-HCl (pH 8.0), 137 mM NaCl, 10% glycerol, 1% TritonX-100, 2 mM EDTA and 2 × protease inhibitor (COMPLETE, Roche Diagnostics GmbH., Mannheim, Germany), and 50 μg was separated by SDS-PAGE followed by semi-dry transfer onto a PVDF membrane. The subsequent immunodetection followed a standard protocol with the primary antibody anti- *I. variabilis*-EPSPS polyclonal antiserum (2 mg mL^−1^) used at a 1:1000 dilution and the secondary antibody HRP-conjugated goat anti-rabbit IgG (ABclonal Inc., MA, USA) diluted with a ratio of 1:10,000.

### Agronomic performances of homozygous transgenic lines without glyphosate treatment

In 2014, the agronomic performances of three T_3_ homozygous transgenic lines (ZY21, ZY25, and ZY29) were compared with that of wild type Zhonghua11. The seeds of ZY21, ZY25, ZY29, and wild type Zhonghua11 were sown at the same time in the field. Twenty-four days after sowing, the seedlings were transplanted into the same field site. Three replicated plots were designed for the candidate transgenic lines and wild type Zhonghua11 with a line distance of 14 cm and a row distance of 18 cm in each plot (20 plants per plot). The field layout was arranged in a randomized design. Heading stage was estimated as the time period from sowing to the day when 50% plants showed panicles. At maturity, 10 plants randomly selected from each plot were used to evaluate the plant height, panicles per plant, panicle length, filled grains per panicle, grain filling rate, 1000-grain weight, and yield per plant. Analysis of variance (ANOVA) was carried out to test significant differences of agronomic performances between the four materials. Following ANOVA, means of the agronomic performances of the four materials were further analyzed using least significant difference method (Fisher LSD) at the 5 and 1% probability levels.

### Glyphosate tolerance assay in the field

In 2014, three T_3_ homozygous transgenic lines (ZY21, ZY25, and ZY29) were treated with five different dosages of glyphosate in the field. The five dosages of glyphosate were 0, 840, 1680, 3360, and 8400 g ha^−1^ respectively with the applied concentrations of 0, 840, 1680, 3360, and 8400 mg L^−1^, and each dosage was supplemented with 0.5% (v/v) Tween 20. The treatment with each dosage for each line was performed in three replicated plots both at seedling and tillering stage. The glyphosate application time and rice cultivating method were as follows: about 50 seeds of each transgenic line were sown in each randomly arranged plot (with a size of 0.014 m^2^); 10 d after sowing, the seedlings in each plot were sprayed over with one of the dosages mentioned above for the first time; 14 d later, 20 transgenic plants were randomly selected from each plot and transplanted into the plot in which the line distance and row distance between plants were 14 and 18 cm, respectively; 15 d later, the second glyphosate treatment was performed for each plot with the same glyphosate dosage used at the first time. Heading stage and agronomic performances at maturity were investigated according to the method described above. At anthesis, 10 spikelets with mature pollen grains were randomly selected from 10 plants of each plot. Pollen viability was evaluated by staining the pollen grains with Lugol's Solution. Analysis of variance (ANOVA) was carried out to test significant differences in agronomic performances of each line between the five dosages of glyphosate. Following ANOVA, means of the agronomic performances were compared using least significant difference method (Fisher LSD) at the 5% probability level. In 2015, one T_5_ homozygous transgenic line (ZY21) was tested again with glyphosate treatment in the same way as in 2014, except that the transgenic plants in each plot (20 plants per plot) were grown with a line distance of 18 cm and a row distance of 20 cm. Statistical analysis method in 2015 was same to that used in 2014.

## Results

### Codon optimization of *I. variabilis-EPSPS* and *ctp*

The average GC content of the original nucleotide sequence of *I. variabilis-EPSPS* was as high as 77% (consisting of 11.4% A, 40.8% C, and 36.1% G and 11.7% T). To avoid the adverse effect of the high GC content on the transcription and translation of *I. variabilis-EPSPS*, the nucleotide sequence of *I. variabilis-EPSPS* was optimized without changing the final amino acid sequence, resulting in a decrease of the average GC content to 66.7%. A chloroplast targeting signal peptide (CTP) is needed to target the *I. variabilis*-EPSPS into the chloroplast. The *ctp* of *A. thaliana EPSPS* with a length of 228 bp contains 48.3% GC, but the codon adaptation index (CAI) in monocots is only 0.66. Hence, the original *ctp* was also optimized, resulting in a substantial increase of CAI to 0.98. The optimized *I. variabilis-EPSPS* (I. variabilis-EPSPS^*^) and optimized *ctp* (ctp^*^) were fused together, and the final fused sequence had a CAI value of 0.86 and a GC content of 66.5%.

### Transformation and identification of I. variabilis-EPSPS^*^ positive plants at T_0_ generation

**Figure 1:**
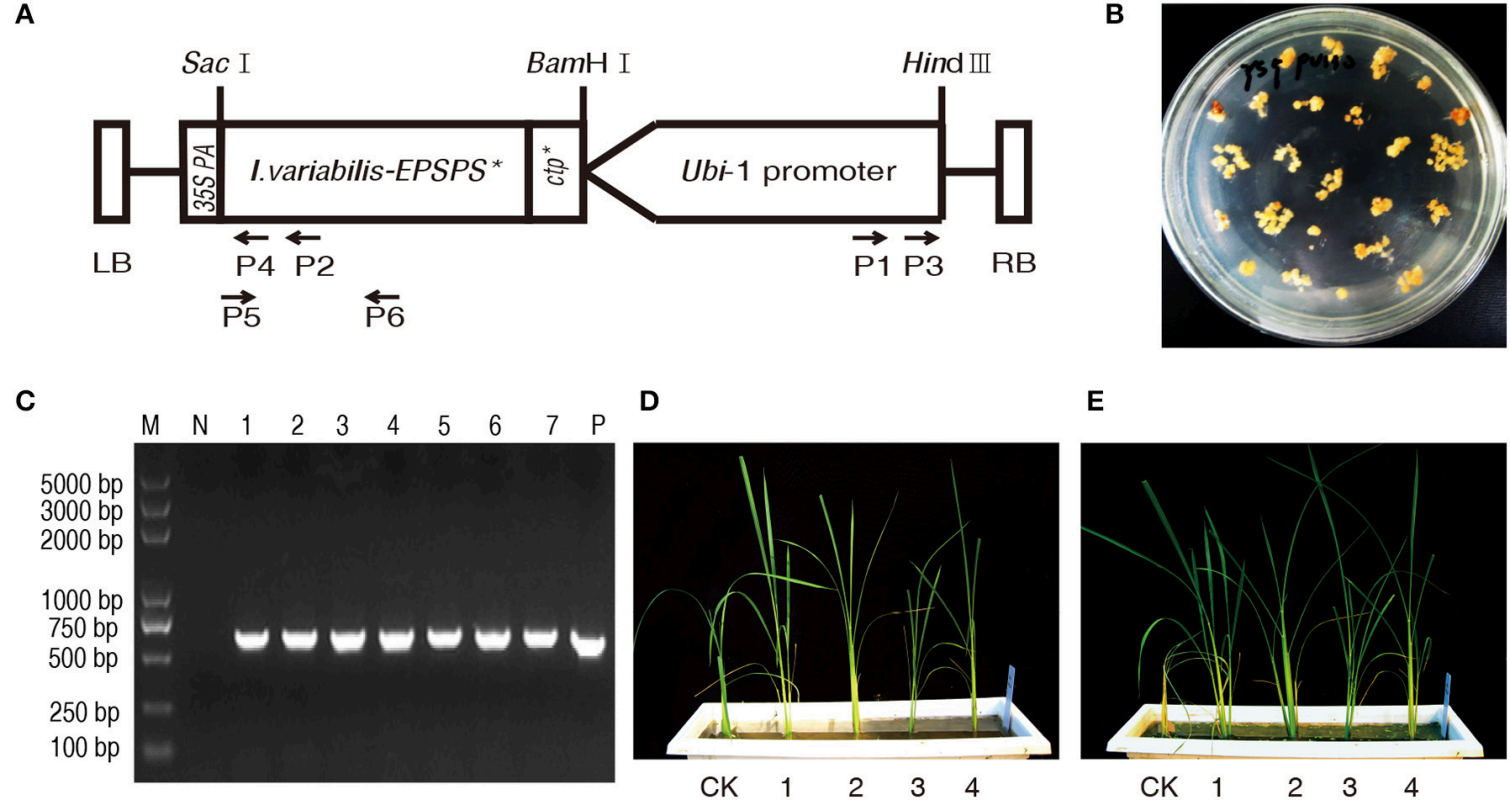
**Transformation and detection of transgenic plants. (A)** In T-DNA region of plant transformation vector of pU130 (*Ubi*-1: I. variabilis-EPSPS^*^: *35S polyA*), I. variabilis-EPSPS^*^ was driven by maize *Ubiquitin*1 promoter and terminated by *35S PolyA*. There was a unique restriction endonuclease recognition site for *Hin*dIII and *Sac*I at the 5′ end of *Ubiquitin*1 promoter and 3′ end of I. variabilis-EPSPS^*^, respectively. P1, P2, P3, and P4 represent primer Ubi-1, IVA-1, Ubi-2, and IVA-2, respectively, which were used to isolate the flanking sequence of T-DNA in the rice genome, while P5 and P6 are primers I. variabilis-EPSPS^*^ -F and I. variabilis-EPSPS^*^-R for PCR assay, by which a DNA fragment with a length of 576 bp was amplified from the transformation vector and I. variabilis-EPSPS^*^ positive lines. **(B)** Obviously resistant calli were formed on the medium containing 200 mg L^−1^ glyphosate 5 weeks after transformation. **(C)** A DNA fragment with the expected size of 576 bp was amplified from positive control pU130 (*Ubi*-1: I. variabilis-EPSPS^*^: *35S polyA*) (lane P) and regenerated rice plants (lane 1 to 7), while there was no DNA fragment amplified from wild type Zhonghua11 (lane N), and lane M represents DNA marker with its band sizes shown beside the lane. **(D,E)** were photographed 0 and 7 d after the glyphosate treatment of T_0_ transgenic plants, respectively. CK represents I. variabilis-EPSPS^*^ negative plant, and 1-4 are I. variabilis-EPSPS^*^ positive T_0_ plants.

With the removal of *hpt* expression cassette, there was only I. variabilis-EPSPS^*^ expression cassette in the final transformation vector pU130 (*Ubi*-1: I. variabilis-EPSPS^*^: *35S polyA*; Figure 1A). So I. variabilis-EPSPS^*^ was used as the selectable marker gene during the *Agrobacterium*-mediated transformation. A large number of obviously resistant calli were obtained after 5 weeks of selection with 200 mg L^−1^ glyphosate (Figure 1B) and a total of 116 independent plants were regenerated from these resistant calli. The regenerated plants were analyzed with PCR. The results showed that 115 of them had an amplified fragment with the expected size (Figure 1C).

To determine glyphosate tolerance, all the 116 independent regenerated lines were sprayed with 3000 mg L^−1^ glyphosate. The I. variabilis-EPSPS^*^ negative plant showed curled leaves 3 d after the treatment and then withered and completely died 7 d later, while over 80% of the I. variabilis-EPSPS^*^ positive plants grew normally without chlorosis in newly-emerged leaves or growth stunting (Figures 1D,E). There were also some I. variabilis-EPSPS^*^ positive plants which were severely inhibited or even killed by glyphosate, which might be due to no or low expression of I. variabilis-EPSPS^*^ in these plants.

### Selection of transgenic plants with a single copy of I. variabilis-EPSPS^*^ integrated into intergenic regions

**Figure 2:**
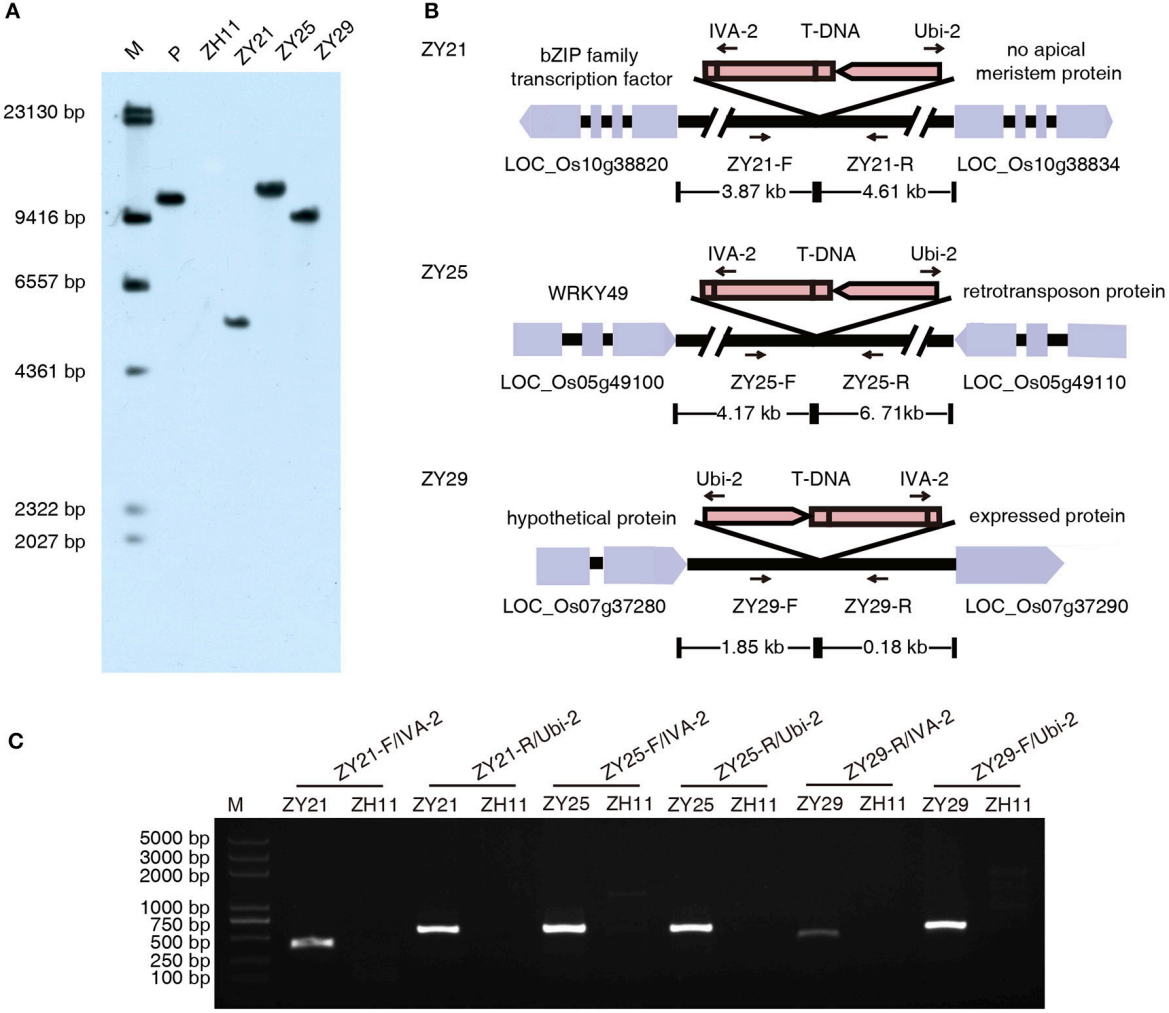
**Southern blot and integration site analysis. (A)** Rice genome DNA was digested with restriction endonuclease *Hin*dIII and detected with DIG-labeled I. variabilis-EPSPS^*^ probe. There was a hybridization band in lane P and lane ZY21, ZY25, and ZY29, which represent positive control pU130 (*Ubi*-1: I. variabilis-EPSPS^*^: *35S polyA*) and the three selected transgenic lines respectively, while there was no hybridization band in lane ZH11 (wild type Zhonghua11). Lane M is DIG-labeled DNA marker with its band sizes shown beside the lane. **(B)** The integration features of T-DNA in ZY21, ZY25, and ZY29 are shown in this diagram. In ZY21, ZY25, and ZY29, the distances of T-DNA integration sites to the upstream and downstream protein coding sequence (CDS) are 3.87 and 4.61 kb, 4.17 and 6.71 kb, 1.85 and 0.18 kb, respectively. ZY21-F, ZY21-R, ZY25-F, ZY25-R, ZY29-F, and ZY29-R are the integration-site specific primers designed according to the sequences nearby the insertion sites, and their sequences are shown in Supplementary Table 1. **(C)** Integration event specific PCR. The primer pair and template for each lane are shown above each lane. M represents DNA marker with its band sizes shown beside the lane.

For commercial release purpose, it is necessary to identify transgene copy number and characterize the transgene locus and the integrity of transgene insertion in plant genome. Thirty-five independent transgenic plants with a relatively high glyphosate tolerance at T_0_ generation were selected and analyzed with Southern blot analysis. Fourteen transgenic plants were confirmed to contain a single copy of I. variabilis-EPSPS^*^. The integration site and the integrity of T-DNA borders in these plants were analyzed by flanking sequence isolation. Three transgenic plants (respectively designated as ZY21, ZY25, and ZY29) were verified to have an intact I. variabilis-EPSPS^*^ expression cassette inserted into intergenic regions. The isolated left border flanking sequences from ZY21, ZY25, and ZY29 were 345, 270, and 128 bp, respectively, and the isolated right border flanking sequences from ZY21, ZY25, and ZY29 were 211, 201, and 190 bp, respectively (Supplementary Data 1). Based on these flanking sequences, there were small insertion-site mutations in the three transgenic lines. For example, a deletion of 20 bp, an insertion of 5 bp undefined sequence, a deletion of 10 bp companied with an insertion of 8 bp undefined sequence existed in ZY21, ZY25, and ZY29, respectively. Integration event specific PCR was further performed with combinations of T-DNA specific primers and of primers designed according to DNA sequences nearby the predicted integration sites. Specific DNA fragments were amplified from the transgenic events ZY21, ZY25, and ZY29 but not from the wild type, which suggested that the predicted integration sites were correct. The results of Southern blot analysis, integration site analysis and integration event specific PCR for the three selected transgenic plants are shown in Figure 2. ZY21, ZY25, and ZY29 were used for subsequent research.

### Selection of T_1_ homozygous transgenic lines

**Figure 3:**
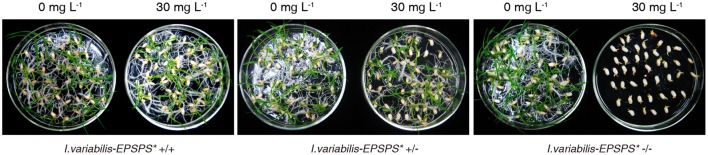
**Selection of homozygous transgenic plants**. Sprouting of the seeds homozygous, heterozygous, and negative transgenic lines on 1/2 MS medium containing 0 or 30 mg L^−1^ glyphosate 7 d after was shown in this figure. 0 and 30 mg L^−1^ represent the concentration of glyphosate for each test.

To investigate the inheritance pattern of the transgene, the seeds from T_1_ transgenic lines were incubated on 1/2 MS medium containing 0 or 30 mg L^−1^ glyphosate. The number of both sprouting seeds and total tested seeds of each line were counted and analyzed via χ^2^ test. Statistical results proved that the progenies of the heterozygous lines were segregated in a ratio of 3:1 on the medium containing 30 mg L^−1^ glyphosate, which indicated that I. variabilis-EPSPS^*^ was inherited in a Mendelian manner (Table 1) and further confirmed the single-copy integration of I. variabilis-EPSPS^*^. Homozygous transgenic lines from ZY21, ZY25, and ZY29 had the same sprouting rate on selective and non-selective medium, while the negative transgenic lines could sprout on the medium containing 0 mg L^−1^ glyphosate but not on the medium containing 30 mg L^−1^ glyphosate (Figure 3). The homozygous transgenic lines of ZY21, ZY25, and ZY29 were selected and used for further research.

**Table 1:** **Segregation of glyphosate tolerance in the progenies of the heterozygous transgenic lines**.

**Heterozygous lines**	**0 mg L**^−1^ **glyphosate**		**30 mg L**^−1^ **glyphosate**		$\chi_{\left(3:1\right)}^{2}$	$\chi_{\left(0.05,1\right)}^{2}$
	**Total tested seeds**	**Sprouting seeds**	**Total tested seeds**	**Sprouting seeds**		
ZY21	500	497	604	444	0.30	3.84
ZY25	287	282	420	315	0.32	3.84
ZY29	507	492	659	473	0.31	3.84

*Sprouting of the progenies of the 3 heterozygous T_1_ lines (ZY21, ZY25, and ZY29) on the medium containing 30 mg L^−1^ glyphosate was tested by Chi-square test. $\chi_{\left(3:1\right)}^{2}$ was calculated with the formula $\chi_{\left(3:1\right)}^{2}\;=\;\frac{{(|A-3\text{a}|-2)}^{2}}{3n}$, in which n is equal to the total tested seeds on the medium containing 30 mg L^−1^ glyphosate multiplied by the ratio of sprouting seeds to the total tested seeds on the medium containing 0 mg L^−1^ glyphosate, A is the number of sprouting seeds on the medium containing 30 mg L^−1^ glyphosate, while a is the number of inhibited seeds which is equal to n minus A*.

### Glyphosate tolerance of T_2_ homozygous transgenic lines on plant medium

**Figure 4:**
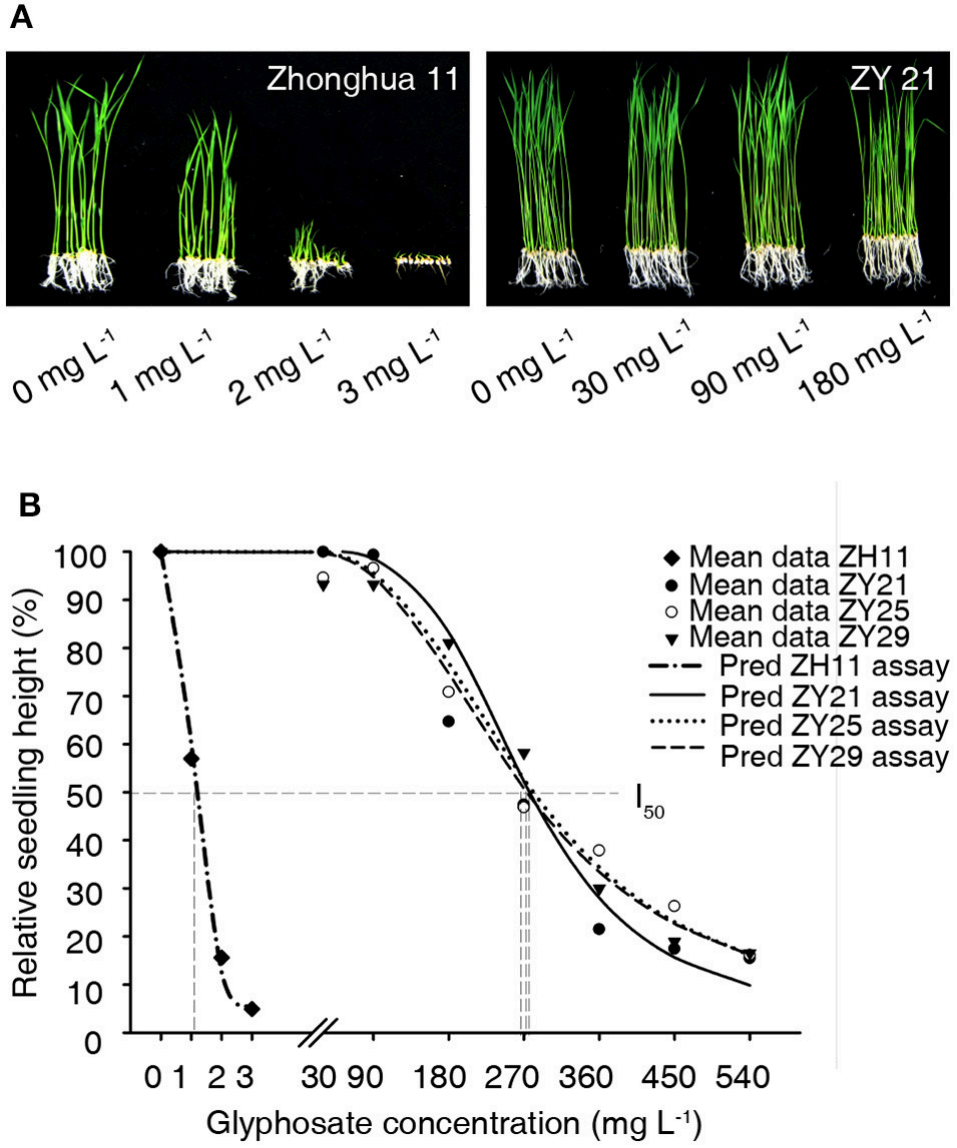
**Glyphosate tolerance assay on plant medium. (A)** The growth status of wild type Zhonghua11 and homozygous T_2_ progenies of ZY21 on the plant medium containing different concentrations of glyphosate 10 d after culturing. The glyphosate concentration for each test is marked with 0, 1, 2, 3, 30, 90, and 180 mg L^−1^. **(B)** Dosage-response curves for wild type Zhonghua11 and the 3 transgenic lines. The predicted curves (Pred ZH11 assay, Pred ZY21 assay, Pred ZY25 assay, Pred ZY29 assay) and mean data points of the relative heights (Mean data ZH11, Mean data ZY21, Mean data ZY25, Mean data ZY29) are plotted in this figure, and the predicted logistic equations for wild type Zhonghua11, ZY21, ZY25, and ZY29 are Y = 3.07 + 96.93/[1 + (X/1.1)^3.72^] [*R*^2^ = 0.98 > $R_{0.01\;\left(3\right)}^{2}$], Y = 3.13 + 96.87/[1 + (X/271.95)^3.79^] [*R*^2^ = 0.93 > $R_{0.01\;\left(6\right)}^{2}$], Y = 2.81 + 97.19/[1 + (X/275.31)^2.73^] [*R*^2^ = 0.98 > $R_{0.01\;\left(6\right)}^{2}$], and Y = 3.11 + 96.89/[1 + (X/266.95)^2.64^] [*R*^2^ = 0.97 > $R_{0.01\;\left(6\right)}^{2}$], respectively. The cross points between the vertical gray dash lines and the X axis represent the I_50_ values for Zhonghua11 and the three transgenic lines.

Wild type Zhonghua11 was very sensitive to glyphosate on the plant medium, as its growth was severely inhibited by just 1 mg L^−1^ of glyphosate and was completely inhibited by 3 mg L^−1^ of glyphosate (Figure 4A). While the homozygous progenies of ZY21, ZY25, and ZY29 grew without visible stunting on the plant medium containing 30 or 90 mg L^−1^ glyphosate (Figure 4A). The dose-response curves for wild type Zhonghua11, ZY21, ZY25, and ZY29 were predicted, in which the I_50_ for each variety was 1.1, 272, 275, and 267 mg L^−1^, respectively (Figure 4B). The results showed that the glyphosate tolerance of these homozygous transgenic progenies was over 240 times that of wild type Zhonghua11.

### Expression of I. variabilis-EPSPS^*^ in homozygous T_3_ transgenic lines

**Figure 5:**
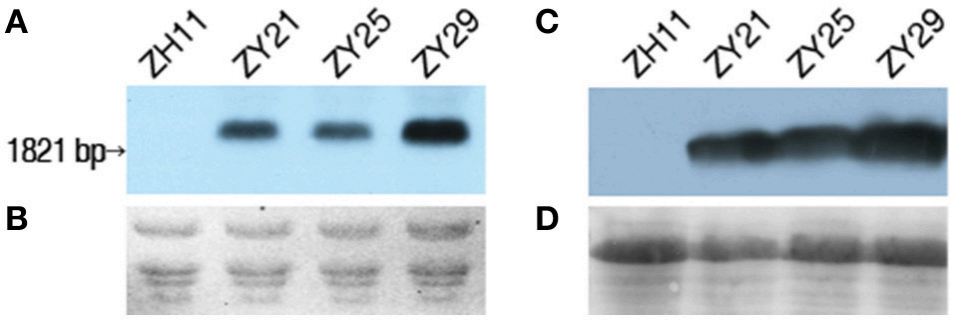
Expression of I. variabilis-EPSPS^*^ in T_**3**_ homozygous transgenic lines. (A) Transcription of I. variabilis-EPSPS^*^ was detected with Northern blot, and there was a hybridization band in lane ZY21, ZY25, and ZY29 but not in lane ZH11. **(B)** RNA loading in each lane corresponds to one lane in **(A)**. **(C)** The translation of *I. variabilis*-EPSPS in wild type Zhonghua11, ZY21, ZY25, and ZY29 was detected with anti- *I. variabilis*-EPSPS polyclonal antiserum, and a specific band was only detected in lane ZY21, ZY25, and ZY29, but not in lane ZH11. **(D)** The protein loading in each lane corresponding to one of the 4 lanes in **(C)** was detected by staining the PVDF membrane with Ponceau. Lane ZH11, ZY21, ZY25, and ZY29 in **(A,C)** represent wild type Zhonghua11 and homozygous T_3_ plants of ZY21, ZY25, and ZY29, respectively.

The expression of I. variabilis-EPSPS^*^ in the homozygous T_3_ transgenic lines of ZY21, ZY25, and ZY29 was confirmed at RNA and protein level with wild type Zhonghua11 being used as the negative control. The three homozygous transgenic lines had a hybridization band with a size corresponding to the expected size in Northern blot (Figures 5A, B), suggesting that I. variabilis-EPSPS^*^ could be normally expressed in these lines, but the expression levels might vary among different lines, which was further confirmed by Western blot (Figures 5C,D).

### Agronomic performances of the selected transgenic lines without glyphosate treatment

In 2014, the agronomic performances of T_3_ homozygous transgenic lines of ZY21, ZY25, and ZY29 were compared with those of wild type Zhonghua11 under the condition without glyphosate treatment. The results indicated that most agronomic performances of one transgenic line ZY21 were statistically not different from those of wild type Zhonghua11, and ZY21 showed better comprehensive agronomic performances than ZY25 and ZY29 (Table 2). It was thus predicated that ZY21 is more suitable for agricultural production than other two transgenic lines.

**Table 2:** **Agronomic performances of homozygous transgenic lines without glyphosate treatment**.

**Rice lines**	**Heading stage (d)**	**Plant height (cm)**	**Panicles per plant**	**Panicle length (cm)**	**Filled grains per panicle**	**Filled grain rate (%)**	**1000-grain weight (g)**	**Yield per plant (g)**
ZY21	70.0 ± 1.0bB	102.9 ± 1.3abA	10.2 ± 1.5aA	22.5 ± 0.2aAB	97 ± 4bA	77.81 ± 1.16aA	21.61 ± 0.47bB	21.11 ± 2.10bAB
ZY25	70.0 ± 1.0bB	98.7 ± 2.2bA	9.6 ± 1.1aA	21.7 ± 0.6bB	82 ± 6cB	76.04 ± 3.67aA	23.73 ± 1.08aA	18.48 ± 3.53bcB
ZY29	73.3 ± 0.6aA	98.1 ± 3.4bA	9.7 ± 0.3aA	23.1 ± 0.3aA	78 ± 5cB	73.00 ± 1.47aA	20.69 ± 0.60bB	15.69 ± 1.18cB
ZH11	69.3 ± 0.6bB	105.3 ± 3.2aA	10.9 ± 0.3aA	22.9 ± 0.2aA	106 ± 2aA	78.59 ± 1.20aA	22.00 ± 0.49bAB	25.42 ± 0.27aA

*Agronomic performances of the wild type Zhonghua11 (ZH11) and the homozygous transgenic lines of ZY21, ZY25, and ZY29 were investigated. The parameters are presented as means (± standard deviation) for data collected from 10 plants in three replicates for each plant type. The lower-case letters and the capital letters in each column represent the differences determined by least significant difference (LSD) method with P < 0.05 and P < 0.01, respectively*.

### Glyphosate tolerance of homozygous transgenic lines in the field

To evaluate the agricultural potential of the selected transgenic lines under glyphosate application, these transgenic lines were treated with different dosages of glyphosate in the field. In 2014, although the homozygous T_3_ progenies of ZY21, ZY25, and ZY29 were treated twice (at seedling and tillering stage) with 840, 1680, 3360, or 8400 g ha^−1^ glyphosate, the major agronomic performances of the treated transgenic plants were not significantly affected compared with those treated with 0 g ha^−1^ glyphosate, except that the panicle number of ZY25 was significantly changed by 3360 and 8400 g ha^−1^ glyphosate treatments (Table 3). As most agronomic performances of all the three transgenic lines were not affected by the treatments with high levels of glyphosate, it was inferred that I. variabilis-EPSPS^*^ has great value in developing glyphosate-tolerant rice. Compared with ZY25 and ZY29, ZY21 showed both better comprehensive agronomic performances and undiminished glyphosate tolerance. Hence, ZY21 was selected to be the final candidate glyphosate-tolerant line.

**Table 3:** **Agronomic performances of the selected transgenic lines under different glyphosate treatments in 2014**.

**Homozygous lines**	**Glyphosate dosage (g ha^−1^)**	**Heading stage (d)**	**Pollen viability (%)**	**Plant height (cm)**	**Panicles per plant**	**Panicle length (cm)**	**Filled grains per panicle**	**Filled grain rate (%)**	**1000-grain weight (g)**	**Yield per plant (g)**
ZY21	0	70.0 ± 1.0a	82.34 ± 3.75a	99.1 ± 2.2a	9.5 ± 0.7a	22.0 ± 0.4a	96 ± 6a	76.10 ± 1.17a	22.36 ± 0.67a	20.28 ± 2.98a
	840	71.0 ± 1.0a	81.36 ± 5.33a	100.6 ± 1.9a	10.1 ± 0.7a	21.9 ± 0.3a	95 ± 4a	77.79 ± 3.00a	22.42 ± 0.36a	21.35 ± 2.12a
	1680	70.7 ± 0.6a	86.01 ± 5.73a	103.3 ± 1.4a	10.3 ± 1.1a	22.0 ± 0.5a	104 ± 4a	79.88 ± 1.63a	22.81 ± 0.54a	24.19 ± 3.71a
	3360	70.3 ± 1.5a	79.18 ± 3.28a	100.2 ± 1.2a	9.0 ± 0.3a	21.5 ± 0.1a	94 ± 5a	73.55 ± 3.07a	21.77 ± 0.39a	18.33 ± 0.95a
	8400	71.7 ± 0.6a	83.04 ± 0.95a	100.1 ± 4.7a	8.8 ± 0.7a	22.4 ± 0.8a	106 ± 10a	79.09 ± 3.00a	23.02 ± 0.65a	21.06 ± 2.95a
ZY25	0	69.7 ± 0.6a	86.98 ± 7.43a	96.8 ± 1.1a	10.2 ± 0.7a	21.2 ± 0.2a	82 ± 6a	77.16 ± 1.05a	23.56 ± 0.94a	19.53 ± 2.01a
	840	70.0 ± 1.0a	80.81 ± 1.03a	97.7 ± 1.2a	9.1 ± 0.6abc	21.4 ± 0.5a	81 ± 9a	75.94 ± 6.77a	23.09 ± 0.38a	16.89 ± 1.03a
	1680	69.7 ± 0.6a	83.82 ± 1.65a	98.9 ± 2.9a	9.7 ± 1.1ab	21.7 ± 0.3a	87 ± 7a	78.68 ± 0.89a	23.37 ± 0.56a	19.32 ± 0.55a
	3360	71.7 ± 1.5a	75.43 ± 2.08a	95.2 ± 1.5a	8.6 ± 1.0bc	21.1 ± 0.4a	82 ± 5a	78.73 ± 3.10a	23.58 ± 1.13a	16.55 ± 1.97a
	8400	71.3 ± 1.2a	83.35 ± 4.39a	95.0 ± 3.1a	8.2 ± 0.4c	21.6 ± 0.5a	89 ± 9a	79.65 ± 3.50a	23.84 ± 1.00a	17.26 ± 1.73a
ZY29	0	72.7 ± 0.6a	75.81 ± 6.74a	94.9 ± 1.3a	10.6 ± 1.5a	22.2 ± 0.5a	76 ± 7a	72.95 ± 3.10a	21.16 ± 0.97a	16.79 ± 1.33a
	840	72.3 ± 0.6a	81.03 ± 5.74a	97.5 ± 1.8a	9.2 ± 0.4a	22.1 ± 0.3a	80 ± 10a	71.17 ± 9.14a	20.97 ± 0.31a	15.20 ± 1.41a
	1680	72.3 ± 0.6a	79.79 ± 11.76a	98.3 ± 2.8a	10.8 ± 0.4a	22.0 ± 0.3a	84 ± 12a	76.66 ± 4.91a	20.91 ± 0.80a	18.86 ± 2.97a
	3360	72.3 ± 0.6a	79.24 ± 1.78a	96.4 ± 2.4a	10.5 ± 0.3a	21.9 ± 0.9a	83 ± 5a	74.55 ± 0.80a	20.68 ± 1.08a	18.14 ± 0.68a
	8400	73.0 ± 2.0a	75.76 ± 2.67a	95.3 ± 5.2a	9.5 ± 1.0a	22.2 ± 0.3a	83 ± 12a	76.04 ± 2.65a	21.83 ± 1.29a	17.15 ± 3.97a

*The parameters are shown as means (± standard deviation) for data collected from 10 plants in three replicates for each treatment. The lower-case letters in each column represent the differences determined by least significant difference (LSD) method with P < 0.05. The field assay was performed in 2014*.

In the glyphosate tolerance assay performed in 2015, although the heading stage of the homozygous T_5_ progenies of ZY21 was 2 days delayed by 8400 g ha^−1^ glyphosate compared with that treated with 0 g ha^−1^ glyphosate, other agronomic performances were not significantly affected by glyphosate treatment (Table 4). The 2-year glyphosate tolerance assay for ZY21 suggested that the glyphosate tolerance of ZY21 was stable and suitable for commercial release.

**Table 4:** **Agronomic performances of ZY21 under different glyphosate treatments in 2015**.

**Glyphosate dosage (g ha^−1^)**	**Heading stage (d)**	**Pollen viability (%)**	**Plant height (cm)**	**Panicles per plant**	**Panicle length length (cm)**	**Filled grains per panicle**	**Filled grain rate (%)**	**1000-grain weight (g)**	**Yield per plant (g)**
0	72.0 ± 1.0a	96.49 ± 2.11a	102.8 ± 3.7a	10.1 ± 0.7a	21.4 ± 0.1a	121 ± 15a	88.86 ± 1.78a	24.41 ± 0.03a	29.32 ± 2.10a
840	72.7 ± 0.6a	95.32 ± 0.92a	105.0 ± 2.1a	10.3 ± 0.4a	21.6 ± 0.7a	112 ± 3a	82.56 ± 3.70a	24.30 ± 1.16a	28.24 ± 2.18a
1680	72.7 ± 0.6a	95.75 ± 0.84a	102.7 ± 0.9a	9.7 ± 1.0a	21.6 ± 1.0a	114 ± 11a	84.50 ± 4.29a	24.04 ± 0.31a	26.76 ± 4.45a
3360	72.7 ± 0.6a	95.76 ± 1.54a	103.3 ± 2.2a	9.4 ± 0.8a	21.4 ± 0.4a	118 ± 10a	82.63 ± 4.37a	24.40 ± 0.74a	27.03 ± 5.36a
8400	74.0 ± 0.0b	94.43 ± 4.07a	103.3 ± 2.2a	8.7 ± 0.4a	21.8 ± 0.3a	129 ± 15a	84.56 ± 6.87a	24.61 ± 0.25a	27.27 ± 4.49a

*The parameters are given as means (± standard deviation) for data collected from 10 plants in three replicates for each plant type. a and b represent the differences determined by least significant difference (LSD) method with P < 0.05. Means in the same column with the same letter are not significantly different from each other. The field assay was performed in 2015*.

## Discussion

Native EPSPSs have been grouped into two classes according to the conserved amino acid sequences and the sensitivity to glyphosate. Usually classI EPSPSs from most plants and Gram-negative bacteria are considered to be sensitive to glyphosate, but with some amino acid mutations they become tolerant to glyphosate as demonstrated by many studies (Comai et al., 1983; Eschenburg et al., 2002; Zhou et al., 2006; Kahrizi et al., 2007; Funke et al., 2009; Tian et al., 2013, 2015). In contrast classI EPSPSs, which comprise EPSPSs from *Agrobacterium tumefaciens* sp. strain CP4, *Achromobacter* sp. strain LBAA, *Pseudomonas* sp. strain PG2982, *Bacillus subtilis, Staphylococcus aureus*, and *Halothermothrix orenii* H168, have intrinsic tolerance to glyphosate and have < 30% homology to classI EPSPSs (Fitzgibbon and Braymer, 1990; Barry et al., 1997; Priestman et al., 2005; Tian et al., 2012). Recently, some novel EPSPSs which are innately insensitive to glyphosate but have conserved sequences close to those of classI EPSPSs have been discovered, including 4G-1, GRG23, and GRG51, AM79 AroA, Gr5_aroA_, and AroA_J.sp_ (Sun et al., 2005; Peters et al., 2010; Cao et al., 2012; Wang et al., 2014; Yi et al., 2016). Although, many glyphosate-tolerant EPSPSs have been discovered, only CP4 EPSPS, maize EPSPS mutant and GRG23 mutant are currently present in ISAAA's GM approval database (http://www.isaaa.org/gmapprovaldatabase/). The amino acid sequence of *I. variabilis*-EPSPS in our study was significantly different from the three EPSPSs, as the amino acid sequence identity of *I. variabilis*-EPSPS with CP4 EPSPS, maize EPSPS mutant and GRG23 mutant is 27.1, 33.5, and 31.5%, respectively (Barry et al., 1997; Alibhai et al., 2010; Peters et al., 2010). The amino acid sequence of *I. variabilis*-EPSPS is also significantly different from that of G6, OsEPSPS mutant, MdEPSPS mutant, VvEPSPS mutant, and AroA_J.sp_, which have been applied in developing transgenic rice (Zhao et al., 2011; Tian et al., 2013, 2015; Chandrasekhar et al., 2014; Yi et al., 2016). Hence, transgenic rice with I. variabilis-EPSPS^*^ will represent a novel type of glyphosate-tolerant rice.

Herbicide-tolerant genes are important selectable marker genes for plant transformation. In previous reports related to glyphosate-tolerant rice, either *hpt* gene or *bar* gene was used as the selectable marker gene, but glyphosate-tolerant *EPSPS* genes were rarely used as the selectable marker gene in rice transformation directly (Tian et al., 2013; Chandrasekhar et al., 2014; Deng et al., 2014; Chhapekar et al., 2015). Although *G6* gene has been used as the selectable marker gene, the PCR positive ratio of the transformants was relatively low and the number of transformants with a single copy of foreign gene was rather limited (Zhao et al., 2011). So far, only *AroA*_*J*.*sp*_ gene has been reported to be highly efficient in rice transformation (Yi et al., 2016). In our research, I. variabilis-EPSPS^*^ was directly used as the selectable marker gene, and the results showed that both the PCR positive ratio of the transformants and the percentage of the transformants containing a single copy of foreign gene were fairly high. Furthermore, we performed an additional experiment to confirm the transformation efficiency with I. variabilis-EPSPS^*^ as the selectable marker gene. We co-cultured the calli of Zhonghua11 with *EHA105* (I. variabilis-EPSPS^*^), which had an OD _600_ value of 0.3. Resistant calli were found in over 50% of the infected calli and the I. variabilis-EPSPS^*^ positive rate of the regenerated plants from these resistant calli was 100% (Supplementary Table 2 and Supplementary Figure 1). The transformation efficiency of using I. variabilis-EPSPS^*^ as the selectable marker gene was comparable to that of using *AroA*_*J*.*sp*_ gene (Yi et al., 2016). Hence, I. variabilis-EPSPS^*^ is a good candidate of selectable marker genes in rice transformation.

Compared with that of wild type Zhonghua11, the glyphosate tolerance of the homozygous transgenic rice (ZY21, ZY25, and ZY29) was greatly improved (by over 240 times) on the medium containing different concentrations of glyphosate. We also determined the glyphosate tolerance of the transgenic rice at much higher glyphosate concentrations. The results showed that they could even sprout on a medium containing 2160 mg L^−1^ glyphosate (equivalent to 1.28 × 10^4^ μM; Supplementary Figure 2), which was significantly higher than that in previous reports (Tian et al., 2013, 2015). Additionally, our data demonstrate that about 50% of T_0_ transgenic plants which contain a single copy of I. variabilis-EPSPS^*^ have such high glyphosate tolerance (Supplementary Figure 3). Thus, the high glyphosate tolerance presented by ZY21, ZY25, and ZY29 is not a special case, and I. variabilis-EPSPS^*^ is suitable for developing transgenic rice highly tolerant to glyphosate.

Glyphosate is a herbicide with a mode of action in which the chemical is translocated from source to sink tissue and inhibits plant meristem growth. Low dosage of glyphosate might not kill sensitive plants immediately but will lead to growth stunting, abnormal leaves, sterility, delayed maturity, and so on (Ye et al., 2001). Such injuries have also been found in the research of the effect of drift rate of glyphosate on non-transgenic rice (Koger et al., 2005). Researches on commercially planted glyphosate-tolerant maize and cotton have shown that even though glyphosate does not cause visible injury to the transgenic crops at the vegetative stage, pollen viability is severely affected at the reproductive stage (Thomas, 2004; Chen et al., 2006; Yasuor et al., 2007). Unfortunately, there have been no reports about the effect of glyphosate on the agronomic performance of transgenic rice. According to our previous research, one transgenic rice line with glyphosate tolerance 60 times that of wild type on the 1/2 MS medium was little inhibited by glyphosate treatment at seedling stage. However, some important agronomic performances of this transgenic line were damaged at late stage. For example yield per plant of this transgenic line was decreased by 840 g ha^−1^ glyphosate, heading stage was several days delayed by 8400 g ha^−1^, and the panicle number per plant was decreased by 1680 g ha^−1^ glyphosate (unpublished data). Therefore, evaluating the glyphosate tolerance of transgenic rice just by visible injury is not enough to prove its feasibility in agricultural production. In previous research, it was reported that 840 g ha^−1^ glyphosate could completely kill non-transgenic rice and weeds in rice paddy (Yi et al., 2016). In this research, most agronomic performances of the candidate transgenic lines were not affected by glyphosate treatments even though the glyphosate dosage was as high as 8400 g ha^−1^. Hence, the selected transgenic lines in our research have the potential to be used in agricultural practice to control weeds with glyphosate. The combination of these transgenic lines and glyphosate can greatly promote the development of direct seeded rice by simplifying the weed control.

Agronomic performances of the selected transgenic crops are important for commercial production. But unintended changes of the plant phenotype not caused by the expression of the transferred gene but by insertional mutagenesis or somaclonal variations are frequently observed during transformation (Bregitzer et al., 1998; Barrell and Conner, 2011; Zhang et al., 2014). In our research, to reduce the possibility of the insertional mutagenesis, we analyzed the insertion site of I. variabilis-EPSPS^*^ firstly. But the selected transgenic lines (ZY21, ZY25, and ZY29) with transgene inserted into intergenic regions still showed different levels of variations compared with the wild type. Such variations might be caused by somaclonal variations or altered expression of some neighboring genes at the insertion site (Latham et al., 2006). Further research has to be performed to analyze the reason for such variations. We finally selected ZY21 as the candidate transgenic line, because ZY21 showed more similar agronomic performances to wild type Zhonghua11 than ZY25 and ZY29. Backcross of ZY21 to wild type can be performed to improve the agronomic performances of ZY21.

Stable integration and expression of the transferred gene are prerequisites for commercialization (Mehrotra and Goyal, 2013). In our research, the molecular characterization of I. variabilis-EPSPS^*^ expression cassette was carefully performed with Southern blot, inverse PCR, Northern blot and Western blot. The results confirmed that only a single copy of intact I. variabilis-EPSPS^*^ was stably integrated into the candidate transgenic lines and I. variabilis-EPSPS^*^ was expressed stably in these transgenic lines. Glyphosate tolerance assay of the candidate transgenic lines was performed in different generations, and the results confirmed that the glyphosate tolerance of the candidate transgenic lines was high and could be transmitted faithfully to the subsequent generations. Hence, the selected transgenic lines, especially ZY21, have great potential to be used in commercial production.

## Author contributions

YL conceived the experiments. YC conducted the experiments. SH gave help in conducting the experiments. ZL and SY cloned *I. variabilis-EPSPS* from the bacterium *Isoptericola variabilis*. FZ, HC, and YL monitored the experimental work. YC and YL analyzed the results and wrote the paper.

### Conflict of interest statement

The authors declare that the research was conducted in the absence of any commercial or financial relationships that could be construed as a potential conflict of interest. The reviewer AD-P and handling Editor declared their shared affiliation, and the handling Editor states that the process nevertheless met the standards of a fair and objective review.
