# Supplementary material for: Development of Novel Glyphosate-Tolerant Japonica Rice Lines: A Step Toward Commercial Release
Source: Front Plant Sci. 2016 Aug 30;7:1218. doi: 10.3389/fpls.2016.01218 (PMC5003930; doi:10.3389/fpls.2016.01218)
Supplement: Supplementary file 1 [file Presentation1.pdf]

Supplementary Material

Development of novel glyphosate-tolerant *japonica* rice lines: a step towards commercial release

Ying Cui[1], Shuqing Huang[1], Ziduo Liu[2], Shuyuan Yi[2], Fei Zhou[1], Hao Chen[1], Yongjun Lin[1*]

[1]National Key Laboratory of Crop Genetic Improvement and National Center of Plant Gene Research, Huazhong Agricultural University, Wuhan, China

[2]National Key Laboratory of Agricultural Microbiology, Huazhong Agricultural University, Wuhan, China

[*]Correspondence:
Yongjun Lin
yongjunlin@mail.hzau.edu.cn

Supplementary Data 1

ZY21 LB flanking sequence

TCGAGTTTCTCCATAATAATGTGTGAGTAGTTCCCAGATAAGGGAATTAGGGTTCCTATAGGGTTTCGCTCATGTGTTGAGCATATAAGAAACCCTTA
GTATGTATTTGTATTTGTAAAATACTTCTATCAATAAAATTTCTAATTCCTAAAACCAAAATCCAGTACTAAAATCCAGATCCCCCGAATTAATTCGGCG
TTAATTCAGTACATTAAAAACGTCCGCAATGTGTTATTAAGTTGTCTAAGCGTCAATTTGTTTACACCAC*AATGGCAACAGTGAAACCTGTCCC*
ACGCCCGGACAGATCCATCCATCTCGCCACCACGCCCCCGTCTCCGCCCAAAACCACCCCAGTCCCCCTCACTATCGAGC
CGCGCCACACGGCGTCGCCGCGCGCGGACGTCGTGACGACGCTTCTCCTCTCCTCTCCTCTTCACCACACGACGCGAC
GCCGACGCCGACGGGGGACGCACGCACGCGCGCGCGCGTACATATGGCGCACGCACGGTCGCGGCACGGCACACG
GCTTGATTTGTGGCCGCGTCGCTGTCTGT-AAAGTCTCTTTTGGGATATCAATCGGAGAGCGCGATACGTACGTACGCGAGG
CA

ZY25 LB flanking sequence

TCGAGTTTCTCCATAATAATGTGTGAGTAGTTCCCAGATAAGGGAATTAGGGTTCCTATAGGGTTTCGCTCATGTGTTGAGCATATAAGAAACCCTTAGTATGT
ATTTGTATTTGTAAAATACTTCTATCAATAAAATTTCTAATTCCTAAAACCAAAATCCAGTACTAAAATCCAGATCCCCCGAATTAATTCGGCGTTAATTCAGT
ACATTAAAAACGTCCGCAATGTGTTATTAAGTTGTCT*AAGCACATTGAGTTTACGCAAACCATTCTTGCAGGACGATCTCGGATAATGGA*
CAGTTCGTACGTAGTAGTAGTACGTTGGGATAAAATTTGGCAATCGAACAATCACTTTCCAGCACTCGCGTCTCACTCACCCT
CACCTCCACATTCATTAATGCTCGATAGGTGATGGTACTACTACTAGAAGATCCCACCCTTGCAACTGCAAGATATCCACTGA
TGACCAATTCATCCACCACTAATTACTCATCTCTACAGGCTCATGTCGTCG

ZY29 LB flanking sequence

TCGAGTTTCTCCATAATAATGTGTGAGTAGTTCCCAGATAAGGGAATTAGGGTTCCTATAGGGTTTCGCTCATGTGTTGAGCATATAAGAAACCCTTA
GTATGTATTTGTATTTGTAAAATACTTCTATCAATAAAATTTCTAATTCCTAAAACCAAAATCCAGTACTAAAATCCAGATCCCCCGAATTAATTCGGCG
TTAATTCAGTACATTAAAAACGTCCGCAATGTGTTATT*AAAAAGCGAAAGTGCAAAACCGCCTCACAAAAACCATCAGCCGCAATGGC*
CGAAACCACCTCCCCTTCGCTTCCCCGCTACAAATACCGCCCCGCCTCCCCCATTCTCCCATCTCCGAAACCCATCAT

ZY21 RB flanking sequence

AAGCTTGGCACTGGCCGTCGTTTTACAACGTCGTGACTGGGAAAACCCTGGCGTTACCCAACTTAATCGCCTTGCAGCACATCCCCCTTTCGCCAG
CTGGCGTAATAGCGAAGAGGCCCGCACCGATCGCCCTTCCCAACAGTTGCGCAGCCTGAATGGCGAATGCTAGAGCAGCTTGAGCTTGGATCAA
ATTGTCGTTTCCCGCCTTCAGTTTAAACTATCAGTGTTTG*CTGTAGCGTCAGCTTTCTCTTGATGTGCCCCAGGAGTGTCGGCGAAAA*
AGGTCAAGACGTTGCATGGCCTAACTGTCGCTATAGGAGGCCCAAAAGCACGGAAGGCAATTTTGAGCCGTGGCCTGTGG
GCTGATTTTAGCAAAGTAAAGGCCCAATAAGATAGTAGTAACTTGAGCGGCCCATCAAACAACCTCACGTGAGAAACAGCAG
A

ZY25 RB flanking sequence

AAGCTTGGCACTGGCCGTCGTTTTACAACGTCGTGACTGGGAAAACCCTGGCGTTACCCAACTTAATCGCCTTGCAGCACATCCCCCTTTCGCCAGCTGGCGTA
ATAGCGAAGAGGCCCGCACCGATCGCCCTTCCCAACAGTTGCGCAGCCTGAATGGCGAATGCTAGAGCAGCTTGAGCTTGGATCAAATTGTCGTTTCCCGCCTT
CAGTTTAAACTATCAGTGTTTGA*TCGCATCGAACATATAAAAGGATGTAGAGAAAAAAAAGATGAGCATGATGACAGTCCAGGCCTG*
AATTTCAGGCTTCGTTAATTCCAAAGTGGGTTTTGAAACCGATTATTAGCCGGACGCAAAACAAGAAAATGATTAATATAGAAT
TAATTAAGTATTACTCCCTCCGGTTTCATTTTAATTGATGCTTTGAGCAATGT

ZY29 RB flanking sequence

AAGCTTGGCACTGGCCGTCGTTTTACAACGTCGTGACTGGGAAAACCCTGGCGTTACCCAACTTAATCGCCTTGCAGCACATCCCCCTTTCGCCAGCTGGCGTA
ATAGCGAAGAGGCCCGCACCGATCGCCCTTCCCAACAGTTGCGCAGCCTGAATGGCGAATGCTAGAGCAGCTTGAGCTTGGATCAGATTGTCGTTTCCCGCCTT
CAGTTTAAACTATCAGTGTTTAAAAACGT*CCCCACCTCCTCCTCTCAGCTGGGACCTGTACACATGTTTTCTCCCGCCATCTCGCCCT*
CCTTCTCGCGAGACGACGACGACGCAGAAACGGATCAGTTTATACTAGTAGTAGTACAGTTTAGCCAACTTTGACATCGAAT
TCTCGTGCCGATATTATCGTGGGAGGAGGAGAAAATGCGGCTACTGTGC

Sequences with yellow background represent 35S polyA terminator; sequences with blue background represent the left border sequences; sequences with green background represent the right border sequences; blue font sequences represent sequences of undefined origin; red italic font sequences represent rice genomic sequences.

Supplementary Table 1

Supplementary Table 1 Primers for integration event specific PCR

Primers	Sequences
ZY21-F	5’- CGAGATGGATGGATCTGTCC-3’
ZY21-R	5’- CTGCTGTTTCTCACGTGAGG-3’
ZY25-F	5’- ACGACGACATGAGCCTGTAG-3’
ZY25-R	5’- TGTAGAACGTGACATTGCTC-3’
ZY29-F	5’- GTAGGCTGTAGCACAGTAGC-3’
ZY29-R	5’- CTACCGATGATGGGTTTCGG-3’

Supplementary Table 2

Supplementary Table 2 Statistical analysis of transformation efficiency

Replicates	*Agrobacterium* concentration (OD_{600})	Number of calli inoculated	Number of resistant calli	Resistant calli rate（%）	Number of regenerated plants	Transgenic positive rate (%)
Ⅰ	0.27	136	79	58.1	22	100
Ⅱ	0.36	158	89	56.3	9	100
Ⅲ	0.32	108	57	52.8	21	100

Supplementary Figure 1

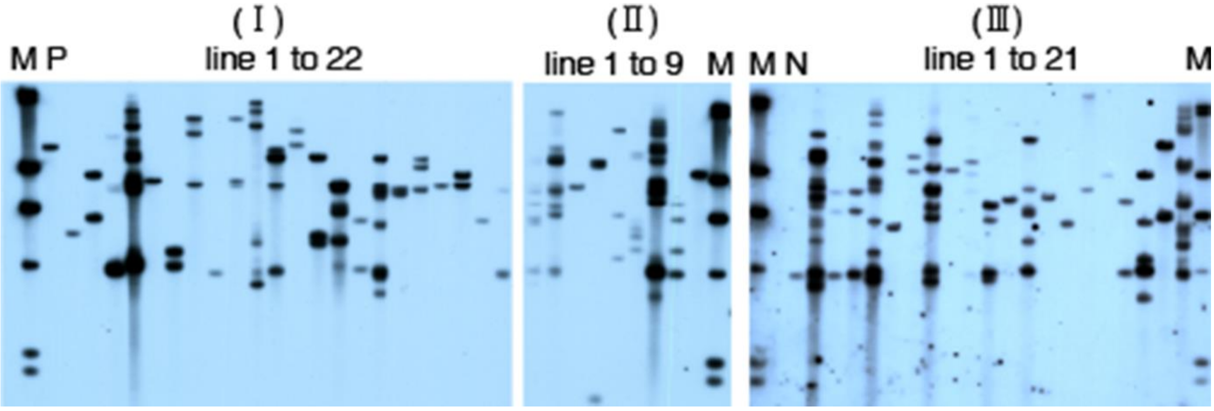

Supplementary Figure 1. Southern blot assay of independent transgenic plants in the three replicates of transformations. 22, 9 and 21 independent transgenic plants respectively obtained from the three independent transformations (Ⅰ, Ⅱ, Ⅲ) were assayed with Southern blot. Rice genome DNA was digested with restriction endonuclease *Hind* Ⅲ and detected with DIG-labeled *I. variabilis-EPSPS** probe. There was a hybridization band in lane P (positive control) and all the detected transgenic plants, while there was no hybridization band in lane N (wild type Zhonghua11). Lane M is DIG-labeled DNA marker with its band sizes 23130 bp, 9416 bp, 6557 bp, 4361 bp, 2322 bp and 2027 bp from the upside to the downside.

Supplementary Figure 2

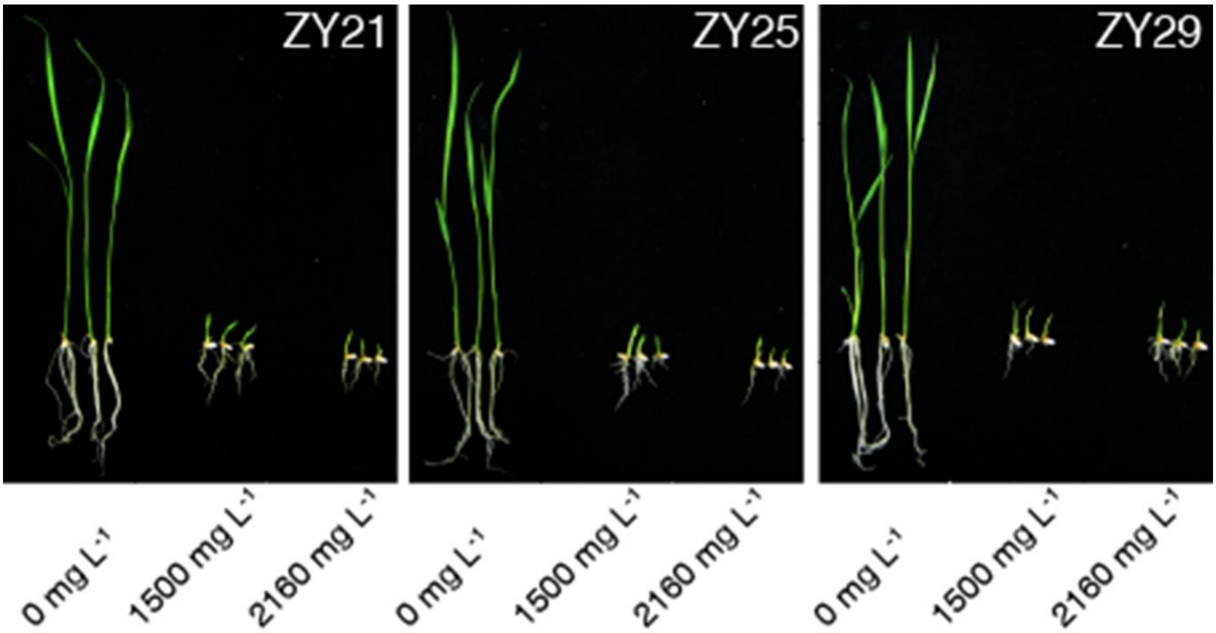

Supplementary Figure 2. Sprouting of transgenic plants on the medium containing high concentrations of glyphosate. T_3 homozygous transgenic progenies of ZY21, ZY25 and ZY29 were geminated on the 1/2 MS medium containing 0, 1500 and 2160 mg L^{-1} glyphosate. 10 d later, although the growth of all the transgenic plants were inhibited on the medium containing 1500 mg L^{-1}, and 2160 mg L^{-1} glyphosate, they still could sprout.

Supplementary Figure 3

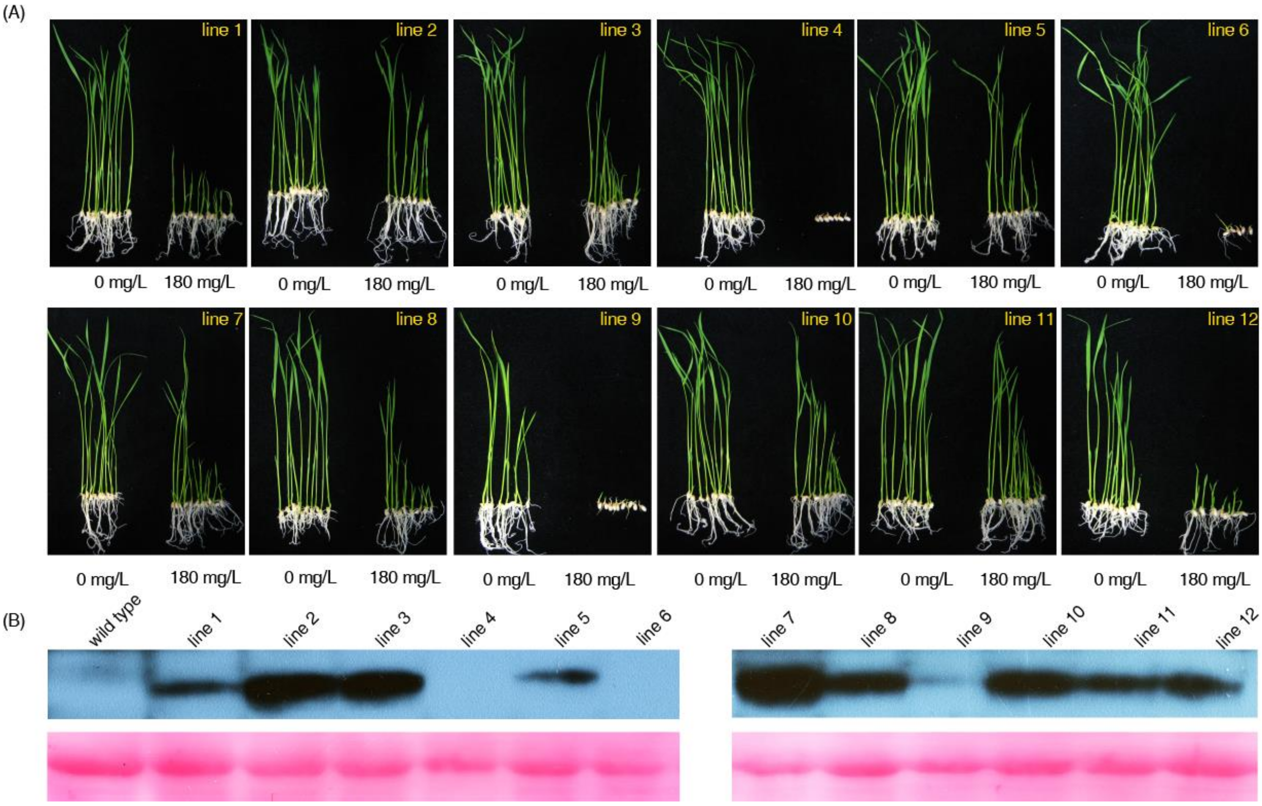

Supplementary Figure 3. The T_1 progenies of 12 independent transgenic plants containing a single copy of *I. variabilis-EPSPS** (the Southern blot analysis was as shown in Supplementary Fiugure S2) were geminated on the 1/2 MS medium containing 0 or 180 mg/L glyphosate. Three transgenic plants (line 4, 6 and 9) were severely inhibited by glyphosate. Western blot assay proved that the expression level of *I.variabilis-EPSPS** in the three transgenic plants was significantly lower than that in the other transgenic plants. 7 lines (line 2, 3, 5, 7, 8, 10, and 11) show similar glyphosate tolerance to ZY21 (the glyphosate tolerance of ZY21 was as shown in the main text), indicating that more than 50% of the transgenic plants containing single copy of *I.variabilis-EPSPS** are highly tolerant to glyphosate.
